# Waist Circumferences of Chilean Students: Comparison of the CDC-2012 Standard and Proposed Percentile Curves

**DOI:** 10.3390/ijerph120707712

**Published:** 2015-07-09

**Authors:** Rossana Gómez-Campos, Cinthya Lee Andruske, Jefferson Hespanhol, Jose Sulla Torres, Miguel Arruda, Cristian Luarte-Rocha, Marco Antonio Cossio-Bolaños

**Affiliations:** 1 Instituto de Ciencias de la Actividad Física y Salud, Universidad Autonoma de Chile, Talca 5 Poniente 1670, Chile; 2 Department of Research, Universidad Científica del Sur, Lima Panamerica Sur Km 19 Villa, Peru; 3 Iberoamericana Research Network of Human Development, Arequipa Urb. Amauta C-6, Jose Luis Bustamante y Rivero, Peru; E-Mail: candruske@gmail.com; 4 Faculty of Physical Education, State University of Campinas, Campinas, Avenida Érico Veríssimo, 701, Cidade Universitária Zeferino Vaz, Barão Geraldo, CEP 13.083-851, Brazil; E-Mail: mcossio30@hotmail.com; 5 Engineering Systems. National University of San Agustin, Catholic University of Santa Maria, Arequipa Urb. San José s/n Umacollo, Peru; E-Mail: rpcaf@gmail.com; 6 Faculty of Physical Education, State University of Campinas, Campinas Avenida Érico Veríssimo, 701, Cidade Universitária Zeferino Vaz, Barão Geraldo, CEP 13.083-851, Brazil; E-Mail: miguel@hotmail.com; 7 Faculty of Physical Sciences Activities, University of San Sebastian, Concepción General Cruz n 1577, Chile; E-Mail: pesquisadores2010@gmail.com; 8 Department of Physical Activity Sciences, Catholic University of Maule, Talca Av. San Miguel 3605, Chile; E-Mail: mcossio1972@hotmail.com

**Keywords:** waist circumference, children, adolescents, Chile, percentiles

## Abstract

The measurement of waist circumference (WC) is considered to be an important means to control overweight and obesity in children and adolescents. The objectives of the study were to (a) compare the WC measurements of Chilean students with the international CDC-2012 standard and other international standards, and (b) propose a specific measurement value for the WC of Chilean students based on age and sex. A total of 3892 students (6 to 18 years old) were assessed. Weight, height, body mass index (BMI), and WC were measured. WC was compared with the CDC-2012 international standard. Percentiles were constructed based on the LMS method. Chilean males had a greater WC during infancy. Subsequently, in late adolescence, males showed values lower than those of the international standards. Chilean females demonstrated values similar to the standards until the age of 12. Subsequently, females showed lower values. The 85th and 95th percentiles were adopted as cutoff points for evaluating overweight and obesity based on age and sex. The WC of Chilean students differs from the CDC-2012 curves. The regional norms proposed are a means to identify children and adolescents with a high risk of suffering from overweight and obesity disorders.

## 1. Introduction

Abdominal obesity, also known as belly fat or clinically known as central obesity, is defined as the excess of abdominal fat that produced around the stomach and the abdomen. The distribution of body fat can be described and evaluated by a variety of anthropometric procedures [1]. Some of these include skinfolds, waist circumference (WC), waist/hip index, and relationship of waist to height, among others.

In fact, some studies have demonstrated that the circumference of the waist is considered as an available, simple, and economical anthropometric measurement [2]. This measurement offers relevant information about the distribution of abdominal fat not only in adults [3] but also in children and adolescents [4,5]. Therefore, the WC should be included in the routine physical examination, especially when cardiovascular diseases and Type 2 diabetes are taken into account [6]. These must be considered since in general studies have demonstrated a close relationship with other risks [7,8,9].

From this perspective, appropriate references for assessing abdominal fat of Chilean children and adolescents do not exist except for the study conducted by Ávalos *et al*. [10]. They assessed the WC of children and adolescents ages 6 to 14. However, the information was incomplete because an important segment of adolescents were not included in the study. In that sense, the evaluation of body fat by means of the WC is relevant since it is a sensitive and accurate indicator of obesity in pediatric populations [11]. In fact, in Chile, students are assessed by means of the BMI. The BMI is commonly used to identify overweight and obesity in children and adolescents.

Therefore, based on the current Nutritional Assessment Technique policy for children and adolescents ages 6 to 18 [12], the Chilean Ministry of Health suggests using the CDC-2012 standard [13]. Moreover, to assess abdominal fat, measuring waist circumference is recommended. Its interpretation through the use of American curves, as described by Fernández *et al*. [14], is commonly used to identify overweight and obesity in children and adolescents even though new standards proposed by the CDC-2012 are available [15]. Based on these new references, it is possible to evaluate not only physical growth but also the nutritional status of children, adolescents, youth, and adults, respectively.

Consequently, identifying and classifying children and adolescents at greater health risk, especially those overweight and obese, is important. In doing so, they then may be diagnosed by using international references. However, it is evident that the references would not reflect the desired patterns of body fat, since the cultural and ethnic variation between the populations are different. Therefore, given the need to understand and explore the WC patterns of Chilean students compared to international benchmarks and the absence of a national Chilean standard, this study has the following objectives: (a) compare the values of waist circumference of students from the Maule Region (Chile) with the CDC-2012 as well as other international standards, and (b) propose a specific reference to assess the WC of students from the Maule Region (Chile) based on age and sex.

## 2. Methods

### 2.1. Participants

A cross-sectional study was designed with 3892 students from the urban zone of the Maule Region of Chile (2092 males and 1800 females). Eight schools from elementary to high school were randomly selected. Students ranged in age from 6 to 18 years old. The size of the sample was determined by probabilistic sampling (quotas). All of the students selected were from public schools in the city of Talca, Chile. The majority of the students attending these schools came from the surrounding cities of the province of Talca. Talca is a city and province of Chile. It is the capital of the Maule Region. Talca is located 243 km to the south of Santiago (the capital of Chile). Talca is considered to be an administrative, economic, and cultural center of the region. It is the most important longitudinal or Central Valley city in Chile. The Human Development Index of the Maule Region for 2012 was 0.72. According to the United Nations Development Program, it was 0.78 for the country [16].

Generally, in Chile, student attendance records are used as socio-economic indicators. Private schools belong to the highest socio-economic level, and public (municipal) schools are considered to be average socio-economically.

In order to conduct the study, all school Directors were asked to participate. Once permission was obtained from all eight schools, parents, guardians, and/or teachers of the children and adolescents were invited to a meeting to explain the project to them and the variables to be evaluated during the study. All parents and guardians agreeing to their children’s participation were given informed consent forms to sign granting their permission for the student assessments. Additionally, permission to conduct the research was obtained from the Ethics Committee form the Universidad Autonoma of Chile.

All students whose parents authorized the assessments of their children, students attending the assessments, and those engaging in a physical activity once a week (90 minutes/day) in their respective schools were included in the study. Students with a physical disability, those refusing to be assessed at the time of the evaluation, and students living in rural areas were excluded.

### 2.2. Procedures

Students’ ages were collected from registration records stored at the participating schools. Students’ chronological age to the decimal point was determined by subtracting the birth date from the age at the time of the measurement. All students were grouped into 13 categories by age and at intervals of one year (for example, 6.0 to 6.9 years).

The entire process of collecting anthropometric information was carried out in the eight schools selected. The measurement process occurred during the months of September to December of 2014.

Prior to data collection, the study group GEISADE trained 6 evaluators to measure the anthropometric variables of weight, height, and circumference of the abdomen. This training was given to students from the professional Physical Education Program. Furthermore, quality control of the anthropometric measurements took place by taking two anthropometric measurements of every 10th subject. This made it possible to verify the intra-evaluator and inter-evaluator technical measurement error (TME). In both cases, the values were less than 3%.

The anthropometric measurement protocol standard adopted for the study was the one proposed by the “international working group of anthropometrics” as described by Ross and Marfell-Jones [17].

The students’ anthropometric variables were measured without shoes and the least amount possible of clothing. Weight was measured by using a digital Tanita (United Kingdom, Ltd.) scale with a precision of 1.0 kg. Height was measured with a portable estadiometer (Seca Gmbh & Co. KG, Hamburg, Germany) with a precision of 0.1 mm. in keeping with the Plan of Frankfurt. The circumference of the waist was measured on top of the skin with a metal metric measuring tape between the lower ribs and the iliac crest. Body mass index (BMI) was obtained from weight and height by using the formula proposed by Quetelet (BMI = weight (kg) /height (m)^2^).

### 2.3. Statistical Analysis

The Kolmogorov-Smirnov (K-S) test confirmed the distribution of the raw data. Descriptive statistics of the arithmetic mean and standard deviation were developed for each age and gender. Comparisons between both sexes were calculated using the t test for independent samples. The t test for paired samples was used to make comparisons between the subjects and the CDC-2012 [15]. Smoothed percentile curves were created for the WC for each sex based on the LMS method [18]. LMS Chart Maker Pro Version 2.3 software [19] was used. The final percentile curves were the result of smoothing three age-specific curves: L (lambda; skewness), M (Mu; median), and S (sigma; coefficient of variation). The following percentiles were calculated: p5, p10, p15, p25, p50, p75, p85, p90, and p95. For all cases, the significance was less than 5%. Comparisons with other international references were depicted graphically using percentile 50 as well as comparisons for percentiles 85 and 95, respectively. The analyses were conducted with SPSS 16.0 for Windows with a level of significance of 5%.

## 3. Results

Table 1 below illustrates the anthropometric variables of weight, height, BMI, and waist circumference for the sample of students studied in this project. In general, the WC of both sexes increased with age. Males compared to females showed greater WC from 6 to 9 years of age and from age 15 to 18 years (*p* < 0.05). However, between the ages of 10 to 15, the values for both males and females were relatively similar.

**Table 1:** Anthropometric characteristics of children and adolescents ages 6 to 18 years from the Maule Region, Chile.

Age (years)	n	Weight (kg)		Height (cm)		Waist Circumference (cm)		BMI (kg/m^2^)	
		Mean	SD	Mean	SD	Mean	SD	Mean	SD
Males									
6	78	26.8	7.0	123.5	6.1	60.2	7.8	17.4	3.5
7	73	30.1	7.5	126.7	7.3	64.6	10.2	18.6	3.8
8	95	36.4	8.0	133.1	6.8	68.5	8.4	20.1	3.2
9	107	38.0	8.8	136,1	9.4	70.8	10.3	19.9	3.6
10	108	42.0	9.1	132.7	14.8	71.7	9.0	20.5	3.3
11	136	48.4	10.9	144.2	17.8	72.5	11.0	21.4	3.6
12	193	52.3	10.5	156.7	11.0	72.3	9.9	21.4	3
13	284	57.1	11.3	161.5	8.7	75.4	10.0	21.9	3.4
14	284	63.2	13.7	167.5	7.2	79.2	11.0	22.5	4.2
15	260	65.0	12.0	169.5	12.6	79.9	9.7	22.5	3.5
16	205	68.7	13.6	171.3	6.0	81.4	11.4	23.4	4.4
17	187	70.1	12.4	172.5	5.9	81.7	10.1	23.5	3.8
18	82	71.9	13.6	172.3	7.4	81.7	9.6	24.2	4.2
Females									
6	81	26.0	5.7	123.2	7.9	56.7	7.8 *	17.1	2.9
7	85	30.1	6.9	126.7	7.0	62.8	8.1 *	18.6	2.7
8	90	31.8	7.2	128.5	6.1	65.4	8.5 *	19.1	3.2
9	97	36.7	9.2	137.5	8.0	66.8	9.6 *	19.3	3.8
10	94	42.5	9.9	142.7	7.8	69.6	10.3	20.7	3.6
11	108	47.4	10.8	149.4	7.4	71.0	10.5	21	3.6
12	128	53.2	13.0	154.5	6.4	72.6	8.9	22.3	5.6
13	164	55.6	9.8	155.9	6.0	74.2	9.5	22.8	3.6
14	241	57.8	9.3	157.0	6.3	76.4	8.4	23.5	3.7
15	204	57.6	9.9	157.4	5.1	76.0	8.5 *	23.2	3.6
16	244	59.9	10.7	159.1	5.5	75.6	8.8 *	23.6	3.8
17	171	59.4	10.3	159.1	6.3	75.6	8.5 *	23.4	3.6
18	93	60.1	9.6	160.1	6.0	75.6	7.5 *	23.4	3.5

Notes: SD: Standard deviation, * (*p* < 0.05): Significant difference in relation to males of the same age.

**Figure 1:**
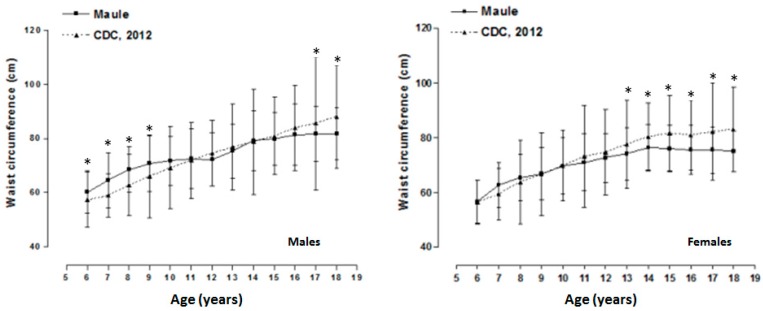
Comparison of averages of waist circumference measurements for the Maule Region (Chile) and the CDC-2012 international index. * Significant difference *p* < 0.05.

Figure 1 illustrates the significant differences between the CDC-2012 references and those found in this study. Males from this research (Maule Region, Chile) showed a greater WC from 6 to 9 years of age in comparison to the international standard (*p* < 0.05). However, during adolescents, the values were relatively similar. Then, at ages 17 and 18, the males from Talca maintained relatively constant WC measurements. As a result, they showed measurements well below the references (*p* < 0.05). For the females from age 6 to age 12, the WC values were similar to the international standard. Subsequently, during all of adolescence (13 to 18 years of age), females showed lower values (*p* < 0.05) in WC.

**Figure 2:**
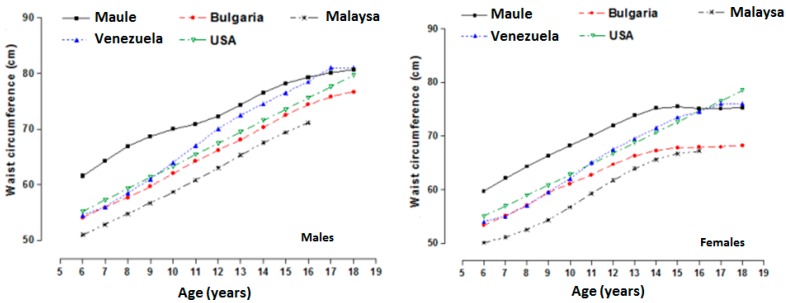
Comparison of the p50 percentile of students of both sexes from the province of Talca (Chile) with other international references.

Figure 2 depicts the percentile (p50) comparisons between the study sample and the international references. Figure 2 shows that both sexes of children and adolescents from the Maule Region (Chile) exhibited greater WC values than those of the international references for children and adolescent of Bulgaria [2], Malaysia [20], the United States [14], and Venezuela [21]. However, the WC reference values of the students of Talca at the end of adolescence were similar to those of students in Venezuela and the United States. Table 2 show the constructed reference curves for WC values for children and adolescents from the Maule Region (Chile) based on age and sex.

**Table 2:** Smoothed curve percentile distribution of waist circumference for children and adolescents of the Maule Region (Chile) based on age and sex.

Age (years)	L	M	S	P5	P10	P15	P25	P50	P75	P85	P90	P95
Males												
6	−0.52439	6.155.318	0.140661	49.4	51.8	53.5	56.1	61.6	67.8	71.6	74.4	78.8
7	−0.55103	6.434.286	0.139546	51.8	54.3	56.0	58.7	64.3	70.9	74.8	77.7	82.2
8	−0.58833	6.685.606	0.138349	54.0	56.5	58.3	61.1	66.9	73.6	77.7	80.6	85.4
9	−0.65335	6.872.915	0.137032	55.7	58.2	60.0	62.8	68.7	75.6	79.8	82.8	87.7
10	−0.75198	6.997.597	0.135455	57.0	59.4	61.3	64.1	70.0	76.9	81.2	84.3	89.3
11	−0.87856	7.094.686	0.1335	58.1	60.5	62.3	65.1	70.9	77.9	82.2	85.4	90.5
12	−102.779	7.225.754	0.130902	59.5	61.9	63.6	66.4	72.3	79.3	83.6	86.9	92.2
13	−119.085	7.428.609	0.127672	61.6	64.0	65.7	68.4	74.3	81.4	85.8	89.1	94.6
14	−137.599	7.651.446	0.123782	64.0	66.3	68.0	70.7	76.5	83.6	88.1	91.5	97.2
15	−161.832	782.086	0.118974	66.0	68.2	69.9	72.5	78.2	85.2	89.7	93.2	99.0
16	−194.069	7.933.414	0.113281	67.6	69.8	71.4	73.9	79.3	86.2	90.6	94.1	100.0
17	−229.912	8.010.295	0.106921	68.9	71.1	72.4	74.9	80.1	86.7	91.0	94.4	100.3
18	−266.586	8.068.186	0.100333	69.8	72.2	73.1	75.8	80.7	86.9	91.1	94.4	100.3
Females												
6	0.11684	5.968.906	0.14066	47.2	49.7	51.6	54.3	59.7	65.6	69.0	71.3	75.0
7	−0.06848	6.214.301	0.13893	49.5	52.1	53.9	56.6	62.1	68.3	71.8	74.3	78.2
8	−0.24588	6.434.521	0.1372	51.6	54.2	55.9	58.7	64.3	70.7	74.4	77.0	81.2
9	−0.39868	6.631.717	0.1353	53.5	56.1	57.8	60.6	66.3	72.8	76.6	79.4	83.7
10	−0.509	6.820.444	0.13291	55.4	57.9	59.6	62.5	68.2	74.8	78.7	81.5	86.0
11	−0.57003	7.007.619	0.12978	57.2	59.8	61.4	64.3	70.1	76.7	80.6	83.5	88.0
12	−0.59462	7.198.154	0.12588	59.2	61.7	63.3	66.3	72.0	78.5	82.5	85.3	89.8
13	−0.63148	7.381.331	0.12145	61.3	63.6	65.4	68.1	73.8	80.3	84.2	87.0	91.4
14	−0.73154	7.519.916	0.11669	63.2	65.2	67.2	69.7	75.2	81.5	85.4	88.1	92.5
15	−0.92937	754.787	0.11197	64.1	66.0	68.0	70.2	75.5	81.6	85.3	88.0	92.4
16	−121.427	7.512.015	0.10724	64.1	65.9	67.7	69.8	75.1	80.7	84.3	87.0	91.2
17	−153.789	7.503.616	0.10231	63.8	65.7	67.2	69.3	75.1	79.6	83.1	85.7	90.0
18	−187.113	753.231	0.09716	63.6	65.7	66.7	69.1	75.3	78.9	82.2	84.7	88.9

**Figure 3:**
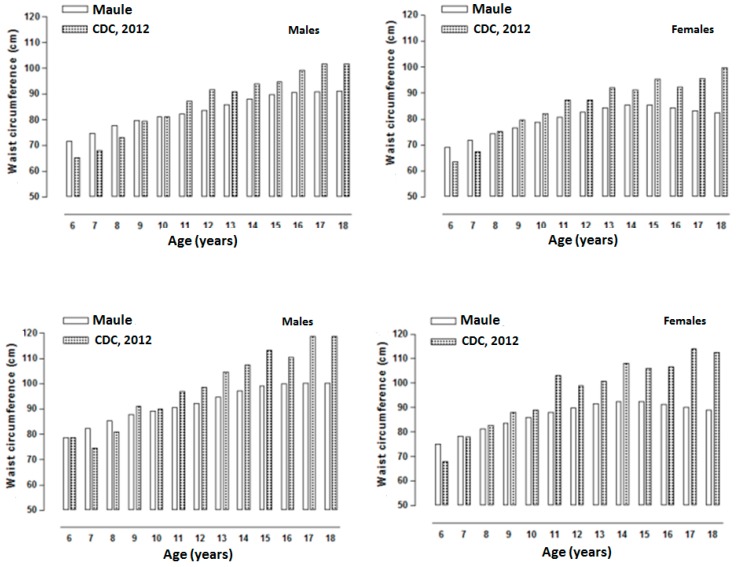
Graphic comparison of percentiles p85 (above) and p95 (below) of the CDC-2012 and the curve of children and adolescents of both sexes from the Maule Region.

Figure 3 illustrates percentiles 85 and 95 of the CDC-2012 and of those in Talca (Chile). For the males, the percentile values were relatively similar until 10 years of age. Then, from ages 11 to 18, the CDC-2012 curves showed higher values than those of the Chilean students. For females, the values in both percentiles (p85 and p95) were relatively similar until 9 years of age. For females from the age of 10, the CDC-2012 curves showed elevated WC values until the age of 18.

## 4. Discussion

Measurement of WC is the simplest and easiest process to carry out. Moreover, this method is non-invasive and economical, and it is widely accepted for assessing the body composition of children and adolescents [22]. The literature has shown that this anthropometric variable correlates with the percentage of fat in the trunk region of the body, independent of place of measurement [23]. Furthermore, it is considered to be an important means to control overweight and obesity in children and adolescents [24].

In this sense, from the WC assessment carried out on the children and adolescents from the Maule Region (Chile), the results of this research show a similar pattern of adiposity (fat) in relation to other studies [2,15,21]. Nevertheless, in absolute terms, significant differences occurred when compared to the international reference of the CDC-2012. In the males of the Maule Region, higher WC values were identified at early ages. Then, the values were relatively similar until 16 years of age. After ages 17 and 18, the international standard shows significantly higher values. On the contrary, for females, mean values were similar until age 12. Later, the females of the Maule Region demonstrated lower mean values compared to the international reference.

The findings obtained from this study simply reflect a greater increase in abdominal obesity in males during infancy compared to the females. In fact, the phenotypic variations identified in the WC of these Chilean students may be due to genetic and cultural factors [2,22]. Students from the Maule Region generally showed higher WC values at all ages in both sexes when compared trans-culturally to international studies. For example, these included comparisons with children and adolescents from Bulgaria [2], children and adolescents from different ethnic groups in the United States [14], children in Malaysia [20], and Venezuela [21]. However, research conducted by Vargas *et al.* [21] and Fernández *et al*. [14] showed relatively similar WC values to those found in the present study for students at ages 16, 17, and 18 in their respective countries.

From that perspective, the high WC values observed during early ages compared to other studies and the lower values found at the end of adolescence related to the CDC-2012 international reference might be conditioned to socio-cultural influences. Some of these may include lifestyle, physical activity, diet, and socioeconomic changes among others. These may play an important role in central body adiposity (fat) in children and adolescents. In fact, this study did not compare for the aforementioned variables that would have allowed for a more comprehensive discussion of the results obtained here. However, it is important to point out that this study included children and adolescents attending municipal schools characterized as socioeconomically average. Furthermore, the sample students only participated in physical activity once a week as a result of physical education classes (90 min/day).

In general, the comparison with other international studies and with the CDC-2012 index should be interpreted with caution due to differences in methodological procedures, differences in the year of data collection [25], and the multiple cultural characteristics of the samples. In fact, it is widely known that children and adolescents from different ethnic populations vary in proportional growth rate and patterns of body fat distribution [26].

Therefore, in light of these discrepancies found compared to international studies and in light of the CDC-2012 international reference, and the lack of a national curve in Chile, the present research study constructed reference curves for WC values for children and adolescents from the Maule Region (Chile) based on age and sex.

To this end, the LMS method was used to construct the regional WC percentiles like various international studies have done [2,20,27]. In fact, this statistical process is widely recommended for evaluating growth, nutritional status, and body adiposity of diverse populations. Furthermore, during the last few years, there has been greater consensus on using percentiles to evaluate overweight and obesity as well as thinness and underweight status of children older than 2 years of age [28].

Thus, among the diverse methods, the LMS method offers some advantages with regard to asymmetric data. Kulaga *et al*. [29] stress that first, extreme percentiles may be estimated more efficiently. Second, whatever percentile is needed may be calculated, and third, each observation may be converted into a numerical standard deviation. The LMS imposes more structure, and it may be more stable in areas where data is scarce. Therefore, its use and application are easy to understand. Furthermore, in practice, it may be used by health professionals and the public in general.

With regard to the cutoff points of the WC, to date, we believe that a consensus does not exist that defines increased risk of adverse health effects for children [30] since multiple explanations exist related to the risk of abdominal overweight and obesity. For example, these variations may be seen in the following percentiles for the different groups: in Spanish children, percentile 75 [31]; in Canadian adolescents, percentiles 90 and 95 [32]; in Malaysian children and adolescents, percentile 90 [20]; and in New Zealand children, percentile 80 [4]. In this context, due to the lack of consensus between the studies, for this research, percentiles 85 and 95 were adopted as appropriate value limits for evaluating overweight and obesity. However, more research is needed to adjust these values and find the ideal limits.

As a result, due to the lack of consensus with regard to the cutoff points, percentiles 85 and 95 were compared graphically with the percentiles of the CDC-2012. The results show that both percentiles are relatively similar at initial ages for both sexes. Subsequently, from ages 9–10, the CDC-2012 percentiles are 5–8 cm greater. Therefore, these values may be overestimated in the distribution patterns of abdominal fat in the students studied in this project. Thus, the percentiles proposed are an alternative to be used provisionally in Chile, especially for students in the Maule Region. However, it cannot be discounted that in the future a greater number of Chilean subjects and cities should be studied. The fundamental purpose of future research would be to generalize these results to the rest of the country.

Our study has several limitations. For example, one is that this was not a probabilistic sample. Moreover, the sample only represented one of the 11 regions of Chile. These disadvantages limit the generalizability to other contexts. Nevertheless, the data from this study provide relevant information for future research. For the first time through this research, percentiles were constructed to assess the WC of children and adolescents in the Maule Region of Chile. These calculations can be seen in the following link: http://reidebihu.net/adiposidad.php.

## 5. Conclusions

The children and adolescents of the Maule Region of Chile differ in terms of WC curves from the CDC-2012 and other international studies. The regional norms proposed provide the possibility to identify children and adolescents at high risk of developing overweight and obesity disorders. These findings may also be a useful tool for monitoring these tendencies in epidemiological and clinical contexts.
